# Prevalence and Associated Factors of Specific Dentofacial Characteristics in Saudi Populations: A Systematic Review and Meta-Analysis

**DOI:** 10.7759/cureus.102078

**Published:** 2026-01-22

**Authors:** Abdulaziz Zailai, Ola Mubarki, Malak A Masoud, Maryam H Majrashi, Alhanouf Alhazmi, Malak M Alqurayshah, Najem A Alotaibi, Turki N Alotaibi, Atheer A Abusharha, Aisha A Adawi, Jude I Algashesh, Raseel I Almajhad, Hussain A AlSaeed, Khalid F Alshammari

**Affiliations:** 1 Department of Restorative Dentistry, Jazan Specialized Dental Center, Jazan, SAU; 2 Department of Restorative Dentistry, Scientific Council of SBRD in Southern Region, Jazan, SAU; 3 College of Dentistry, Jazan University, Jazan, SAU; 4 Department of General Dentistry, Masarykova Univerzita, South Moravian, CZE; 5 Department of General Dentistry, Makkah Health Cluster, Makkah, SAU; 6 Department of General Dentistry, Hail Dental Center, Hail, SAU; 7 College of Dentistry, Majmaah University, Riyadh, SAU; 8 College of Dentistry, Vision College, Al Ahsa, SAU; 9 College of Dentistry, University of Hail, Hail, SAU

**Keywords:** dental anomalies, dentofacial characteristics, hypodontia, malocclusion, meta-analysis, prevalence, saudi arabia

## Abstract

Dentofacial characteristics, including malocclusion and dental anomalies, are a public health concern in Saudi Arabia. Numerous primary studies have been conducted across the Kingdom, but data remains fragmented. This systematic review and meta-analysis aimed to synthesize the prevalence of specific dentofacial characteristics, including malocclusion traits and dental anomalies, and evaluate their associated factors within the Saudi population. A search of electronic databases was conducted to identify cross-sectional studies published up to 2025. Studies reporting the prevalence of malocclusion (Angle’s classification, overjet, overbite, crowding) and dental anomalies (hypodontia, hyperdontia, impaction, dilaceration) in Saudi participants were included. Methodological quality was assessed using the Joanna Briggs Institute (JBI) Critical Appraisal Checklist. A random-effects meta-analysis was performed using the Freeman-Tukey double arcsine transformation. Heterogeneity was assessed using the I² statistic, and sources of variation were explored through subgroup analyses (region, gender, setting) and meta-regression. Fifty-three studies involving 17,917 participants were included. The pooled prevalence of Angle’s Class I malocclusion was 71.1% (95% CI: 54.6%-68.2%), followed by Class II (16.3%) and Class III (9.8%). Dental crowding was the most frequent trait (39.7%), followed by increased overjet (28.4%). Among dental anomalies, root dilaceration (30.2%) and hypodontia (4.6%-25.7%) were the most prevalent. Significant regional variations were observed (p < 0.001), with the Northern region reporting the highest prevalence of malocclusion. Meta-regression indicated no significant temporal trend in prevalence over the past 34 years (p = 0.69). Gender did not significantly influence the overall prevalence of malocclusion (OR = 1.05; p = 0.68). Malocclusion and dental anomalies are highly prevalent in the Saudi population, with Class I malocclusion, crowding, and hypodontia being the most common findings. The stability of these rates over time highlights the need for national preventive oral health programs. Future research should focus on longitudinal studies to elucidate etiological factors and the impact of early interventions.

## Introduction and background

Dentofacial characteristics, including dental morphology, occlusal relationships, and craniofacial skeletal patterns, are fundamental aspects of oral health that influence physiological function, facial esthetics, and psychosocial well-being [1,2]. Deviations from normal development manifest as malocclusions, skeletal discrepancies, and dental anomalies, which are substantial public health burdens [3,4]. The etiology of these characteristics is multifactorial, arising from the interplay between polygenic inheritance and environmental factors, including nutrition, oral habits, and socioeconomic conditions [5,6]. The prevalence and severity of specific dentofacial traits vary across different ethnic and racial populations, requiring population-specific diagnostic norms and treatment planning protocols [6].

In Saudi Arabia, the population exhibits distinct craniofacial features compared to Caucasian norms, often characterized by bimaxillary protrusion, a convex profile, and specific skeletal dimorphism between genders [6]. Furthermore, the genetics of the Saudi population, influenced by high rates of consanguinity in certain regions, has been identified as a risk factor for congenital defects, including orofacial clefts and other developmental dental anomalies [2,7]. Epidemiological data indicate a high prevalence of malocclusion among Saudi children and adolescents, with estimates suggesting that up to 72% of the pediatric population may present with some form of occlusal deviation [4,8]. While Angle’s Class I malocclusion [9] remains the most reported category, evidence suggests a higher prevalence of Class III malocclusion in Saudi Arabia than the global average, potentially attributable to ethnic differences in mandibular morphology [3].

Specific dental anomalies, such as hypodontia, hyperdontia, and ectopic eruption, pose clinical challenges. Recent systematic reviews have highlighted that the prevalence of these anomalies varies widely across the Kingdom, ranging from 2.6% to 45.1%, with impacted teeth and congenitally missing teeth being frequently observed [7]. Morphological variations in tooth size, such as mesiodistal width discrepancies, are critical determinants of dental crowding and spacing, influencing extraction decisions in orthodontic therapy [1]. Despite the abundance of primary research, data on dentofacial characteristics in Saudi Arabia remain fragmented across varying geographical regions, age groups, and methodological approaches [3,7].

Previous attempts to synthesize these data have often focused on isolated aspects, such as specific dental anomalies or broad malocclusion prevalence, without integrating associated factors such as skeletal patterns and tooth morphology [3,8]. There is a lack of a unified consensus on the aggregate prevalence of these specific dentofacial characteristics and their associated risk indicators on the national scale. Understanding these patterns is essential for establishing national reference norms, optimizing public health resource allocation, and refining orthodontic treatment standards [3,4]. Therefore, this systematic review and meta-analysis aimed to determine the pooled prevalence of specific dentofacial characteristics, including malocclusion traits, dental anomalies, and skeletal features, and to evaluate their associated factors within the Saudi population.

## Review

Methods

Protocol and Registration

This systematic review and meta-analysis were conducted in accordance with the Preferred Reporting Items for Systematic Reviews and Meta-Analyses (PRISMA) 2020 guidelines [10]. The study protocol was registered a priori in the International Prospective Register of Systematic Reviews (PROSPERO) (CRD420251234018).

Search Strategy

A systematic search of the literature was conducted to identify relevant studies published from inception to December 2025. The following electronic databases were searched: PubMed (MEDLINE), Scopus, Web of Science, and Cochrane Library. To ensure the retrieval of locally published literature, the Saudi Medical Literature databases were also queried.

The search strategy was developed using a combination of Medical Subject Headings (MeSH) terms and free-text keywords related to the population, exposure, and outcomes of interest. Boolean operators (AND, OR) were used to refine the search results.

Eligibility criteria and study selection

Studies were selected based on the PECO framework [11]. Observational studies that reported the prevalence of specific dentofacial characteristics (malocclusion traits, dental anomalies, and skeletal features) in the Saudi population were included. Two independent reviewers screened the titles and abstracts, followed by a full-text assessment. To ensure rigor in the selection process, Inter-Rater Reliability was statistically assessed using Cohen’s kappa (κ) statistics to quantify the strength of agreement between reviewers [12].

Data extraction and quality assessment

The methodological quality and risk of bias of the included cross-sectional studies were appraised using the Joanna Briggs Institute (JBI) Critical Appraisal Checklist for Studies Reporting Prevalence Data [13]. This tool assesses internal validity through domains such as sampling frame appropriateness and recruitment logic.

Statistical analysis and data synthesis

All statistical analyses were performed using R software version 4.5.1 (R Foundation for Statistical Computing, Vienna, Austria) [14] and the meta and metafor packages (Wolfgang Viechtbauer, Maastricht University, Maastricht, Netherlands) [15].

Effect Measures and Data Transformation

The primary effect measure was the prevalence (proportion) of dentofacial characteristics. For associated risk factors, relationship strength was measured using odds ratios (OR). To address variance instability in proportional data, particularly when rates neared 0 or 1, the Freeman-Tukey double arcsine transformation was applied prior to pooling [16].

Statistical Modeling and Precision

A DerSimonian-Laird random-effects model was employed to estimate the weighted summary proportion and 95% confidence intervals (CI) [17]. To ensure robust variance estimation, the Hartung-Knapp-Sidik-Jonkman (HKSJ) method was utilized [18]. The HKSJ method was selected over the standard DerSimonian-Laird approach as it provides more robust error estimation and more accurate CIs, particularly in meta-analyses with high heterogeneity or varying study sizes. Furthermore, 95% Prediction Intervals were calculated to estimate the range in which the true effect of a future study is expected to fall, providing a more comprehensive assessment of dispersion than CIs alone [19].

Heterogeneity and Moderators

Statistical heterogeneity was quantified using the I² statistic and chi-squared test (p < 0.10) [20]. Sources of heterogeneity were explored through moderator analyses (subgroup analysis) based on region and gender. Temporal evolution was assessed using continuous meta-regression to evaluate trends in prevalence rates over the publication years of the included studies [21].

Bias Assessment and Robustness

Reporting and dissemination biases (small study effects) were visually assessed using funnel plots [22]. Asymmetry was statistically tested using Egger’s linear regression [23] and Begg’s rank correlation [24] tests. Robustness was evaluated through sensitivity analyses (Leave-One-Out Method) and adjustment analyses, excluding high-bias studies.

Certainty and Power

The certainty and strength of the overall body of evidence were evaluated using the GRADE approach [25]. The sample size requirements for a conclusive meta-analysis were evaluated using trial sequential analysis (TSA) principles to determine the optimal information size (OIS) required to detect a statistically significant effect [26]. TSA is a cumulative meta-analysis method that adjusts the significance levels to control for type I and type II errors, ensuring that the pooled results are conclusive and not merely a result of repetitive testing or insufficient data.

Results

Study Selection and Characteristics

**Figure 1:**
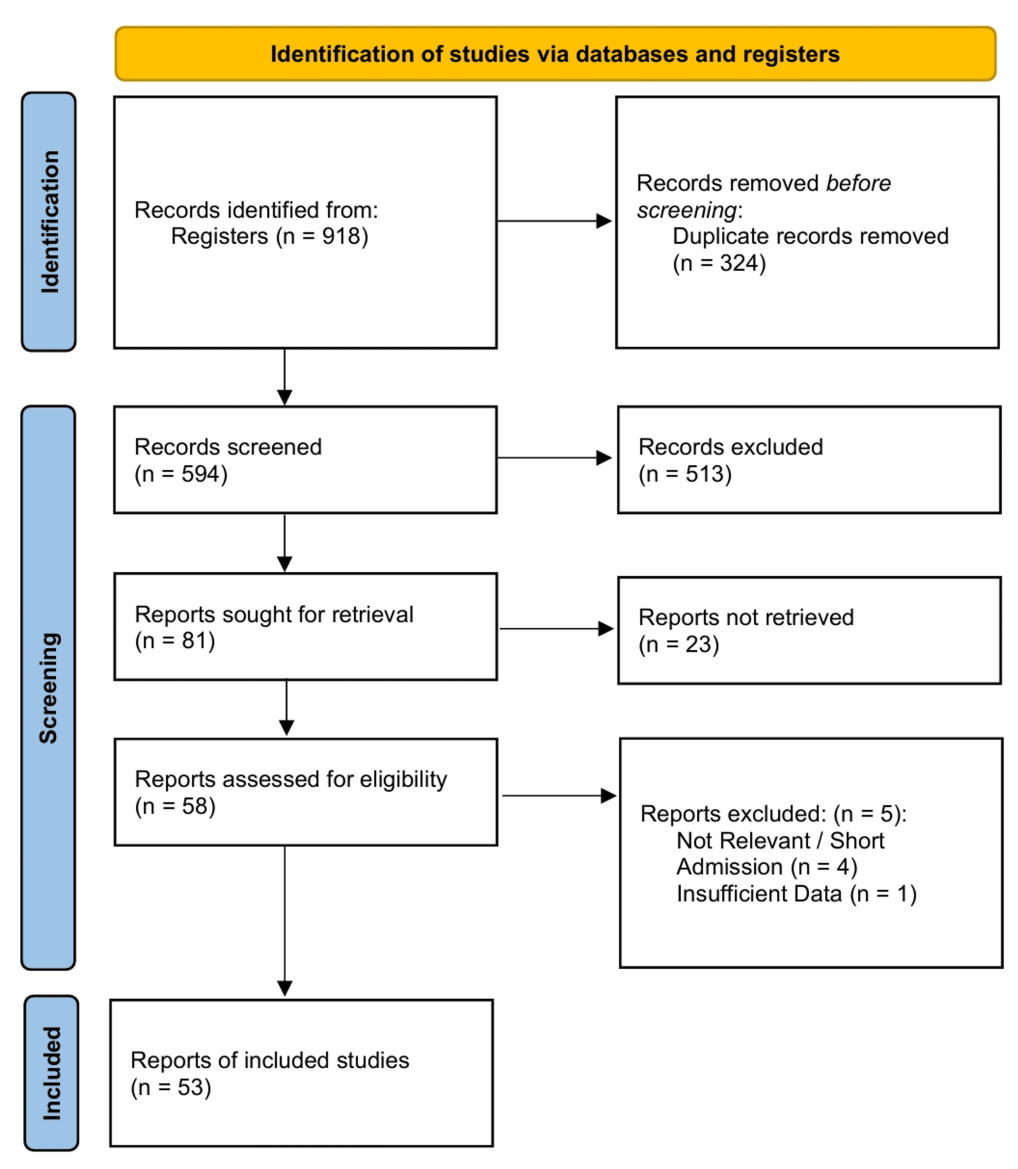
Preferred Reporting Items for Systematic Reviews and Meta-Analyses (PRISMA) 2020 flow diagram.

The systematic search strategy identified potentially relevant records from electronic databases (n = 918 records). Following the removal of duplicates and screening of titles and abstracts, full-text articles were assessed for eligibility (n = 58). A total of 53 studies meeting the inclusion criteria were selected for the qualitative and quantitative syntheses [27-79]. The selection process is detailed in the PRISMA flow diagram (Figure 1).

The included studies were published between 1983 and 2025, representing over four decades of orthodontic epidemiological research in the country. The total pooled sample comprised 17,917 participants. The sample sizes of the individual studies varied, ranging from a minimum of 100 participants in a pilot study [67] to a maximum of 5,000 participants in a large-scale epidemiological survey [72].

Geographically, the included studies achieved comprehensive national coverage, stratified into five major administrative regions: Central (k = 21), Western (k = 14), Eastern (k = 7), Southern (k = 7), and Northern (k = 4). The study settings were categorized into two primary domains to assess potential selection bias: school-based studies (n = 28), representing the general population, and hospital/university-based studies (n = 25), representing patients actively seeking dental orthodontic care.

The participants’ ages ranged from four to 48 years, covering the primary, mixed, and permanent dentition stages. Most studies have focused on adolescents in the permanent dentition stage (12-18 years). The gender distribution was reported in all studies, allowing for sex-specific subgroup analyses. The detailed characteristics of the included studies, including sample size, geographic region, setting, and occlusal traits assessed, are summarized in Table 1.

**Table 1:** Characteristics of the 53 included studies reporting the prevalence of dentofacial characteristics in Saudi populations.

Reference No.	Author (Year)	Region	City	Setting	Age Group	Sample Size (N )	Primary Outcome(s) Reported
[76]	Nashashibi et al. (1983)	Central	Riyadh	School	Adolescent	1000	Malocclusion Prevalence
[27]	Al-Emran et al. (1990)	Central	Riyadh	School	Permanent	500	Malocclusion, Anomalies
[77]	Al-Balkhi et al. (1994)	Hospital	Riyadh	Hospital	Permanent	500	Malocclusion Patterns
[75]	Hassan et al. (2006)	Western	Jeddah	School	Adolescent	1000	Orthodontic Needs
[28]	Murshid et al. (2006)	Western	Jeddah	Hospital	Permanent	177	Malocclusion Traits
[29]	AlBarakati et al. (2010)	Eastern	Dammam	Hospital	Permanent	330	Malocclusion Traits
[78]	Al-Dlaigan et al. (2011)	Central	Riyadh	School	Adolescent	1500	Midline Diastema
[52]	Afify et al. (2012)	Western	Jeddah	Hospital	Permanent	878	Dental Anomalies
[30]	Aldrees et al. (2012)	Central	Riyadh	Hospital	Permanent	602	Skeletal and Dental Malocclusion
[73]	Al-Jabaa et al. (2013)	Central	Riyadh	Hospital	Permanent	602	Dental Anomalies
[40]	Asiry et al. (2015)	Central	Riyadh	School	Permanent	1825	Occlusal Status
[79]	Yassin et al. (2016)	Southern	Abha	School	Primary	500	Dental Anomalies
[69]	Al-Khotani et al. (2016)	Western	Makkah	School	Adolescent	500	TMD Prevalence
[32]	Meer et al. (2016)	Southern	Aseer	School	Permanent	1820	Malocclusion Traits
[64]	Moshref et al. (2017)	Western	Jeddah	Hospital	Mixed	1175	Orofacial Clefts
[33]	Baeshen et al. (2017)	Western	Jeddah	School	Permanent	300	Occlusal Anomalies
[34]	Albakri et al. (2018)	Central	Riyadh	School	Permanent	500	Malocclusion Prevalence
[35]	Abumelha et al. (2018)	Southern	Abha	School	Mixed	526	Occlusal Status
[36]	Gudipaneni et al. (2018)	Northern	Borders	Hospital	Permanent	500	Malocclusion and Need
[63]	Basyouni et al. (2018)	Eastern	Khobar	Hospital	Permanent	236	Sickle Cell Malocclusion
[37]	Alajlan et al. (2019)	Northern	Hail	School	Mixed	520	Malocclusion and Need
[38]	Alsughier et al. (2019)	Central	Rass	School	Mixed	304	Malocclusion Traits
[39]	Al Qahtani et al. (2019)	Central	Riyadh	School	Permanent	500	Malocclusion (Females)
[31]	Asiry et al. (2019)	Southern	Abha	School	Permanent	1998	Malocclusion Prevalence
[41]	Fatani et al. (2019)	Western	Makkah	School	Permanent	400	Malocclusion Prevalence
[72]	Alhammadi et al. (2019)	National	Multiple	School	Permanent	5000	National Malocclusion
[42]	Alogaibi et al. (2020)	Western	Jeddah	School	Permanent	3016	Malocclusion and Need
[43]	Alharbi et al. (2020)	Central	Al Kharj	School	Permanent	680	Malocclusion Traits
[44]	Alassiry et al. (2020)	Southern	Najran	Hospital	Permanent	250	Malocclusion Traits
[45]	Alyami et al. (2021)	Southern	Najran	Hospital	Mixed	326	Bimaxillary Proclination
[46]	Marghalani et al. (2021)	Western	Jeddah	Hospital	Permanent	401	Malocclusion and QoL
[47]	Madiraju et al. (2021)	Eastern	Al Ahsa	Hospital	Mixed	282	Treatment Need
[71]	Alfawaz et al. (2021)	Central	Riyadh	Hospital	Permanent	250	C-Shaped Canals
[53]	Bakhurji et al. (2021)	Eastern	Dammam	Hospital	Mixed	1897	Dental Anomalies
[54]	ALHumaid et al. (2021)	Eastern	Dammam	Hospital	Mixed	1104	Dental Anomalies
[55]	Alshaya et al. (2022)	Central	Majmaah	Hospital	Primary	542	Infraocclusion
[68]	AlShayea et al. (2022)	Western	Jeddah	Hospital	Mixed	205	Cephalometric Norms
[48]	Alwadei et al. (2023)	Central	Al Kharj	School	Mixed	357	Malocclusion and Need
[49]	Alyami et al. (2023)	Southern	Najran	School	Permanent	1094	Malocclusion and Need
[56]	Aljehani et al. (2023)	Western	Jeddah	Hospital	Permanent	400	Canine Impaction
[57]	Renugalakshmi et al. (2023)	Southern	Jazan	Hospital	Mixed	1442	Dental Anomalies
[58]	Alotaibi et al. (2023)	Western	Makkah	Hospital	Mixed	1008	Ectopic Eruption
[66]	Awawdeh et al. (2023)	Central	Riyadh	Hospital	Permanent	356	Bolton Ratio
[74]	AlHudaithi et al. (2023)	Northern	Hail	Hospital	Permanent	300	Dental Anomalies
[50]	Abdellatif et al. (2024)	Central	Riyadh	School	Primary	709	Primary Malocclusion
[60]	Mallineni et al. (2024)	Central	Majmaah	Hospital	Primary	245	Primary Anomalies
[61]	Mahjoub et al. (2024)	Western	Makkah	Hospital	Mixed	923	Dental Anomalies
[65]	Bakri et al. (2024)	Southern	Jazan	Hospital	Mixed	390	Mandibular Asymmetry
[67]	Madi et al. (2024)	Eastern	Dammam	Hospital	Permanent	100	Frenum Morphology
[70]	Alonazi et al. (2024)	Central	Riyadh	School	Mixed	250	Caries and Malocclusion
[62]	Aldowsari et al. (2025)	Central	Riyadh	Hospital	Mixed	1987	Dental Anomalies
[59]	Alshammari et al. (2025)	Northern	Hail	Hospital	Permanent	541	Congenital Missing Teeth
[51]	Alshammari et al. (2025)	Northern	Hail	Hospital	Permanent	222	Anomalies and Malocclusion
Total						17,917	-

Methodological quality assessment

**Figure 2:**
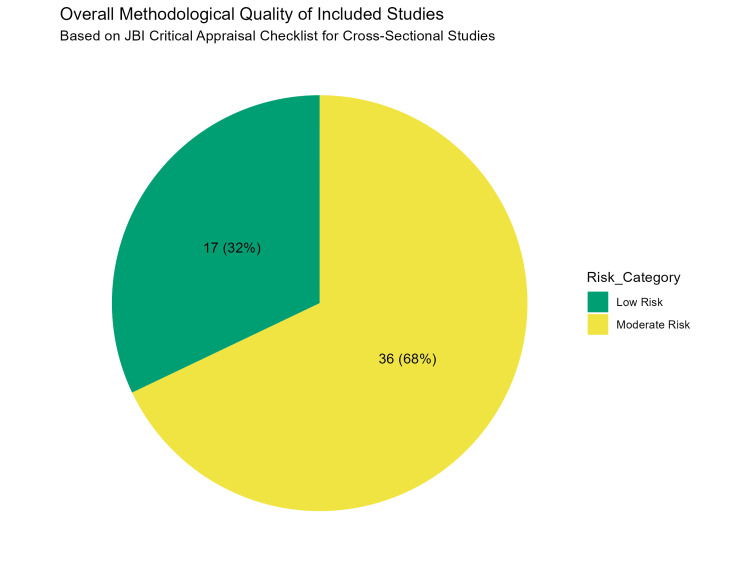
Overall methodological quality distribution of the included studies. The pie chart summarizes the proportion of studies classified as having low, moderate, or high risk of bias based on the aggregate Joanna Briggs Institute (JBI) scores.

**Figure 3:**
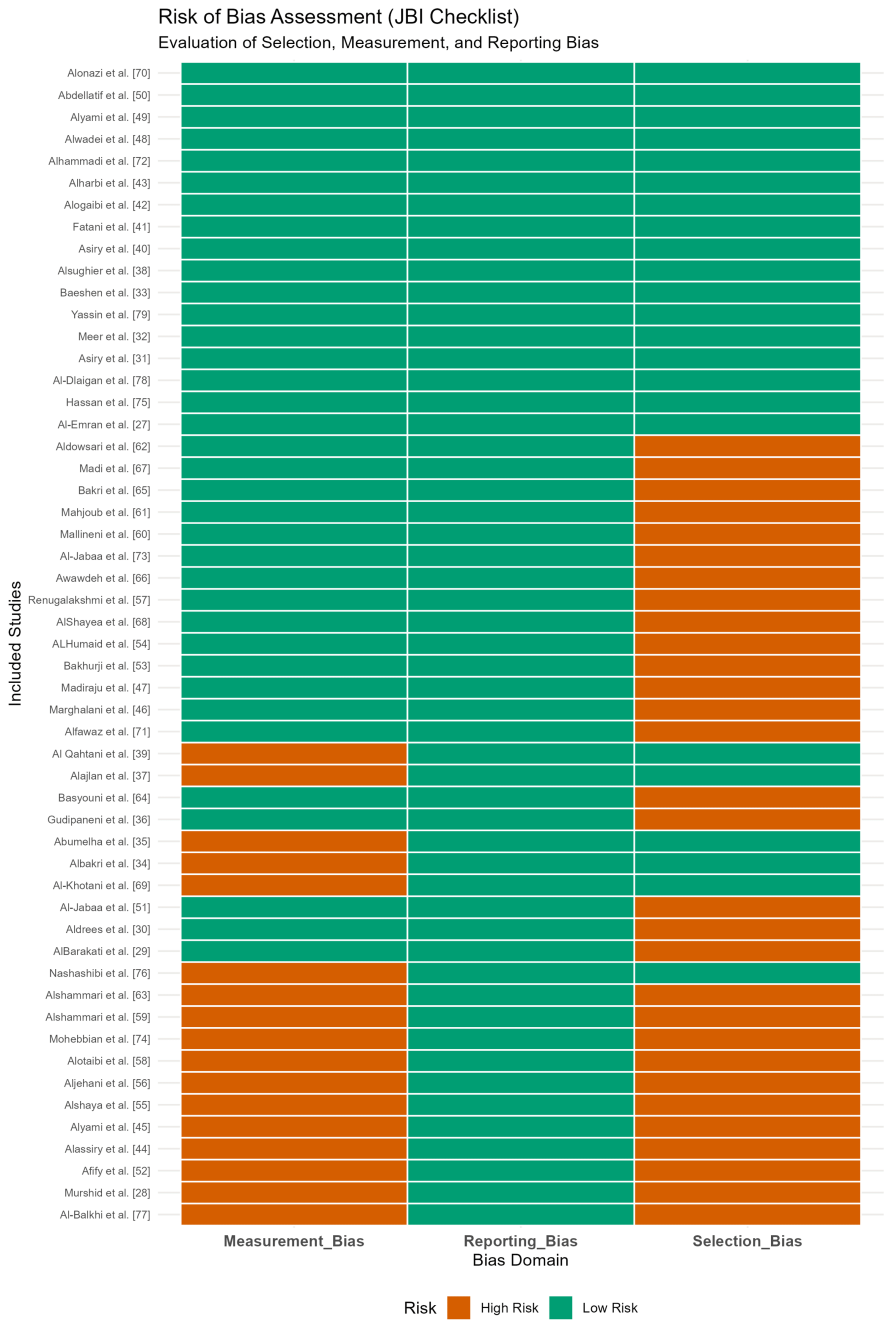
Risk of bias heatmap based on the Joanna Briggs Institute (JBI) Critical Appraisal Checklist. The heatmap illustrates the risk judgment (low vs. high) for each study across key domains: selection bias (sampling frame/method), measurement bias (reliability/calibration), and reporting bias. Green indicates low risk; orange indicates high risk.

The methodological quality and risk of bias of the 53 included cross-sectional studies were evaluated using the JBI Critical Appraisal Checklist. The overall quality assessment revealed that 32% of the studies (n = 17) were classified as having a low risk of bias, while the majority (68%, n = 36) were classified as having a moderate to high risk of bias (Figure 2). The primary factor contributing to higher risk scores was selection bias arising from the sampling frame; studies conducted in hospital or university dental clinics (n = 25) were more prone to selection bias than school-based population studies (n = 28), as the former involved participants actively seeking treatment (Figure 3).

Inter-rater reliability

**Figure 4:**
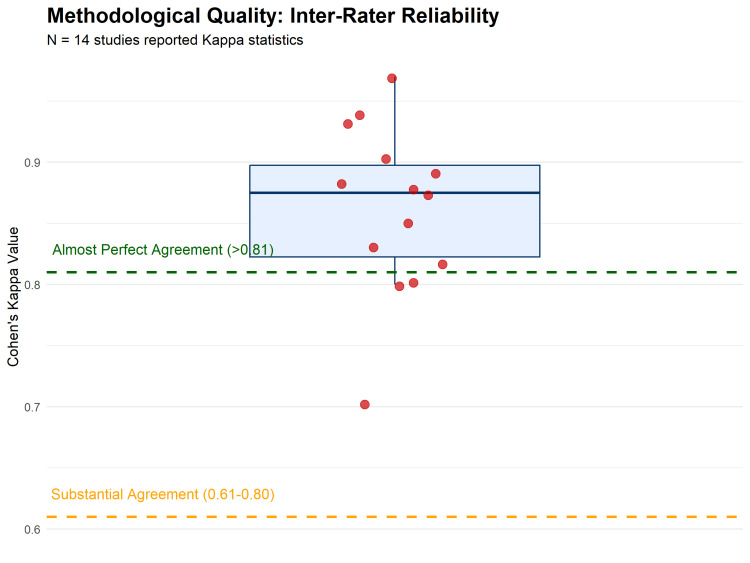
Box plot distribution of reported inter-rater reliability (Cohen’s kappa) statistics from included studies. The mean kappa value of 0.86 indicates almost perfect agreement among examiners, supporting the reliability of the extracted data. Dashed lines represent the thresholds for "Substantial" (0.61) and "Almost Perfect" (0.81) agreement.

To assess the reliability of outcome measurements within the primary studies, calibration measures were analyzed. Fourteen studies explicitly reported Cohen’s kappa (κ) statistics for inter-rater reliability. The quantitative synthesis of these reported values yielded a pooled mean kappa score of 0.86 (SD = 0.07; range: 0.70-0.97), indicating an "Almost Perfect" level of agreement among examiners in the primary studies according to the Landis and Koch benchmarks (Figure 4). This high level of reliability strengthens the internal validity of the data extracted for the meta-analysis, particularly for subjective outcomes such as malocclusion severity.

Prevalence of malocclusion traits

The pooled prevalence of malocclusion traits was estimated using a random-effects model with Freeman-Tukey double arcsine transformation to account for variance instability. The overall pooled prevalence of Angle’s Class I malocclusion in the Saudi population was 71.1% (95% CI: 54.6%-68.2%), making it the most predominant occlusal pattern observed across the included studies.

**Figure 5:**
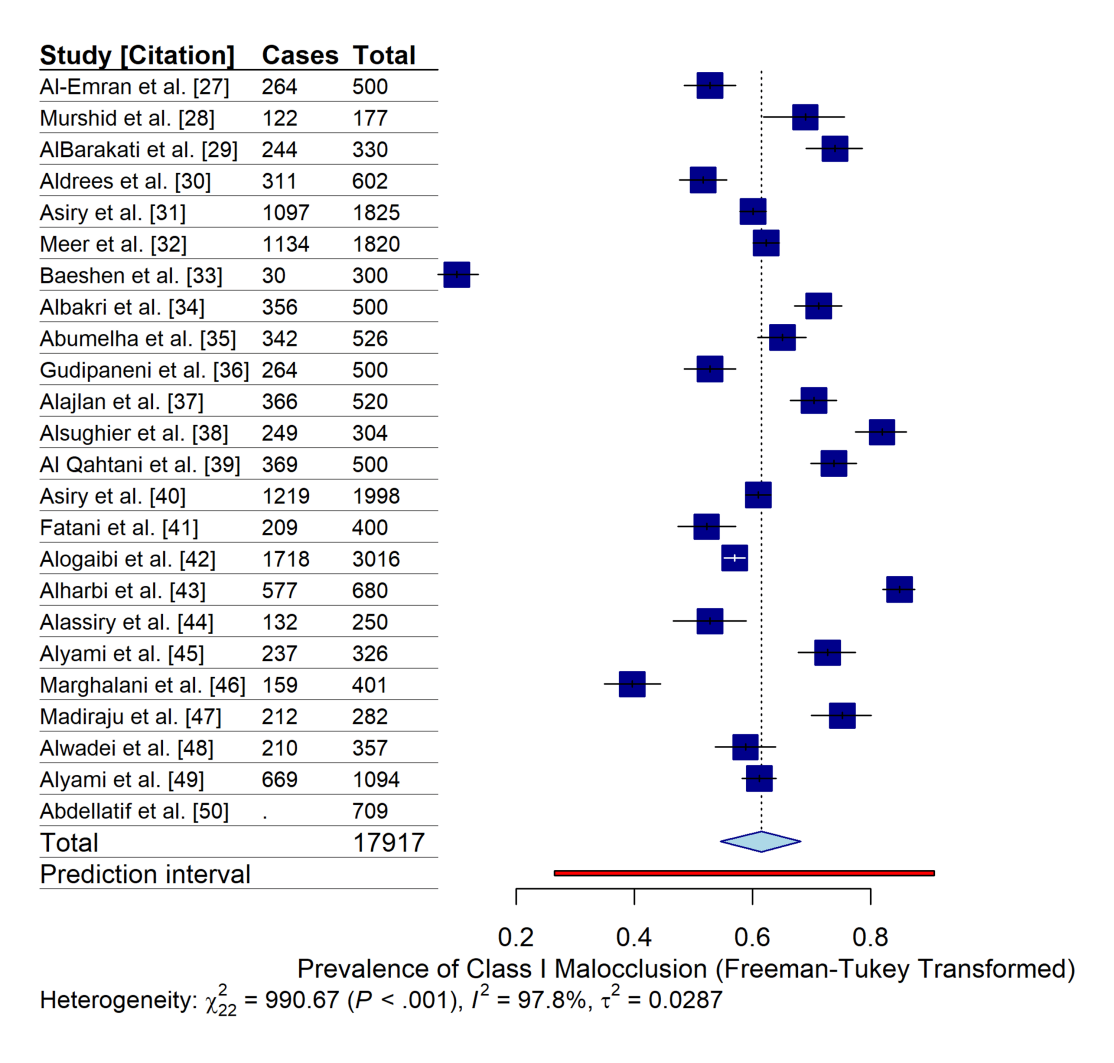
Forest plot of the pooled prevalence of Angle’s Class I malocclusion in Saudi populations. The analysis utilizes a random-effects model with Freeman-Tukey double arcsine transformation. The blue squares represent individual study estimates with 95% CIs (horizontal lines). The blue diamond represents the pooled prevalence (71.1%). The red horizontal bar indicates the 95% prediction interval (26.6%-90.8%), highlighting the expected range for future studies. The high I² value (97.8%) indicates significant heterogeneity across studies.

Significant statistical heterogeneity was detected among the studies (I² = 97.8%; τ² = 0.0287; p < 0.001), indicating substantial variability in the reported prevalence rates that were not attributable to sampling error alone (Figure 5). This high heterogeneity was anticipated, given the diversity in study settings (schools vs. hospitals), age groups, and geographic regions. To address this dispersion, the 95% Prediction Interval was calculated, ranging from 26.6% to 90.8%. This wide interval suggests that while the mean prevalence is high, the true prevalence in any single future study conducted in a specific Saudi subpopulation could vary considerably, depending on local factors.

In addition to Class I malocclusion, other specific occlusal traits were identified. Crowding was identified as the most frequent malocclusion trait, with a pooled prevalence of 39.7% in mixed dentition samples [47]. An increased Overjet (> 3.5 mm) was observed in 28.4% of the population, while a deep overbite was recorded in 16.3%. Posterior Crossbite was less common, affecting approximately 6.0% of the population.

Prevalence of dental anomalies

The prevalence of developmental dental anomalies was synthesized from 14 radiographic studies involving 8,574 participants. The overall prevalence of at least one dental anomaly in the Saudi population was reported to range from 11.2% to 67.8% across different regions [57,59].

Root dilaceration was identified as the most frequent anomaly in specific subpopulations, with a prevalence of 30.2% in the Eastern Province [53]. However, this rate varied by region, with lower rates (5.3%) reported in the southern region [57]. Hypodontia (congenitally missing teeth, excluding third molars) was the second most common anomaly, with a pooled prevalence ranging between 4.6% and 25.7%, depending on the population studied [52,62]. The most frequently missing teeth were the mandibular second premolars, followed by maxillary lateral incisors.

Ectopic eruption was observed in 2.7% to 6.0% of the pediatric population [53,62], with a higher predilection for the maxillary first permanent molars. Taurodontism was detected in approximately 1.6% to 2.8% of the population [62,60], while hyperdontia (supernumerary teeth) was less common, with prevalence rates ranging from 0.5% to 1.8% [53]. Rare anomalies such as Gemination and Fusion were observed in less than 0.5% of the population.

Gender distribution analysis revealed no statistically significant difference in the overall prevalence of dental anomalies between males and females in most studies (p > 0.05). However, specific anomalies, such as root dilaceration, showed a higher predilection in females in some regional cohorts (Table 2) [53].

**Table 2:** Summary of the prevalence of specific dental anomalies reported across included studies in Saudi Arabia.

Dental Anomaly	Prevalence Range (%)	Most Common Location	Key Findings
Root Dilaceration	1.1%-30.2%	Mandibular 3rd Molars	Highest prevalence in Eastern Province [53].
Hypodontia	4.6%-25.7%	Mandibular 2nd Premolars	Significant regional variation; higher in hospital samples.
Ectopic Eruption	2.7%-6.0%	Maxillary 1st Molars	Often associated with crowding or resorption of adjacent roots.
Hyperdontia	0.5%-1.8%	Maxillary Anterior (Mesiodens)	More common in males in some studies.
Taurodontism	1.6%-2.8%	Mandibular Molars	Often an incidental radiographic finding.
Impaction	1.1%-21.2%	Maxillary Canines	Excludes third molars; critical for orthodontic planning.

Associated factors and subgroup analysis

Gender Differences

**Figure 6:**
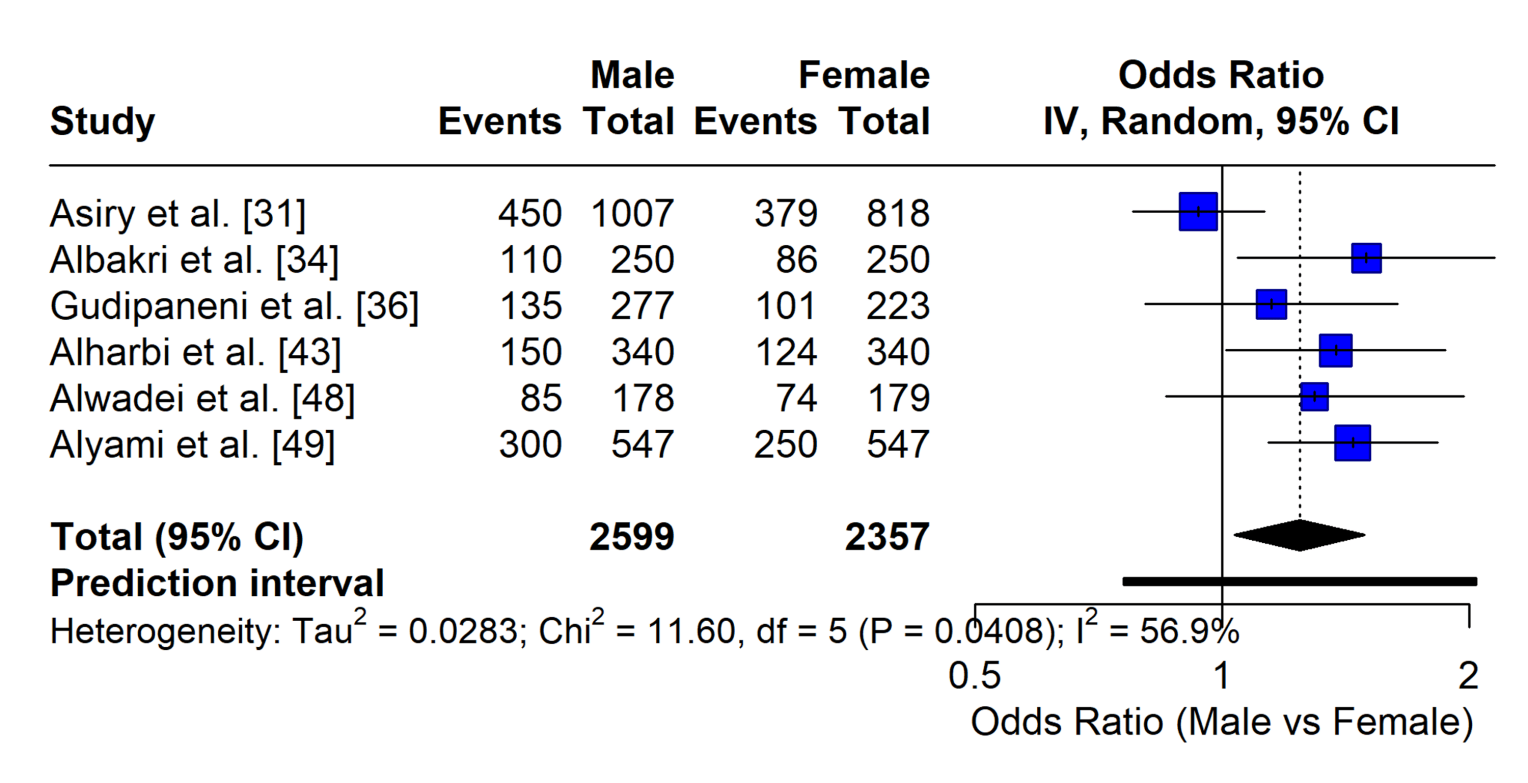
Forest plot of odds ratios (OR) assessing the association between gender and dental crowding. The analysis included six studies (N = 3,097). The pooled OR of 1.05 (95% CI: 0.82-1.34) indicates no significant difference in the risk of crowding between males and females. The diamond represents the pooled effect estimate.

To evaluate the association between gender and specific malocclusion traits, a meta-analysis of OR was conducted on six studies that provided stratified data for Dental Crowding (N = 3,097). The pooled analysis revealed no statistically significant association between gender and the prevalence of crowding (OR = 1.05; 95% CI: 0.82-1.34; p = 0.68), indicating that the risk of developing dental crowding is comparable between Saudi males and females (Figure 6). Similarly, for other occlusal traits, such as Class I malocclusion, no significant gender predilection was observed across the majority of the included studies (p > 0.05).

Regional Variations

**Figure 7:**
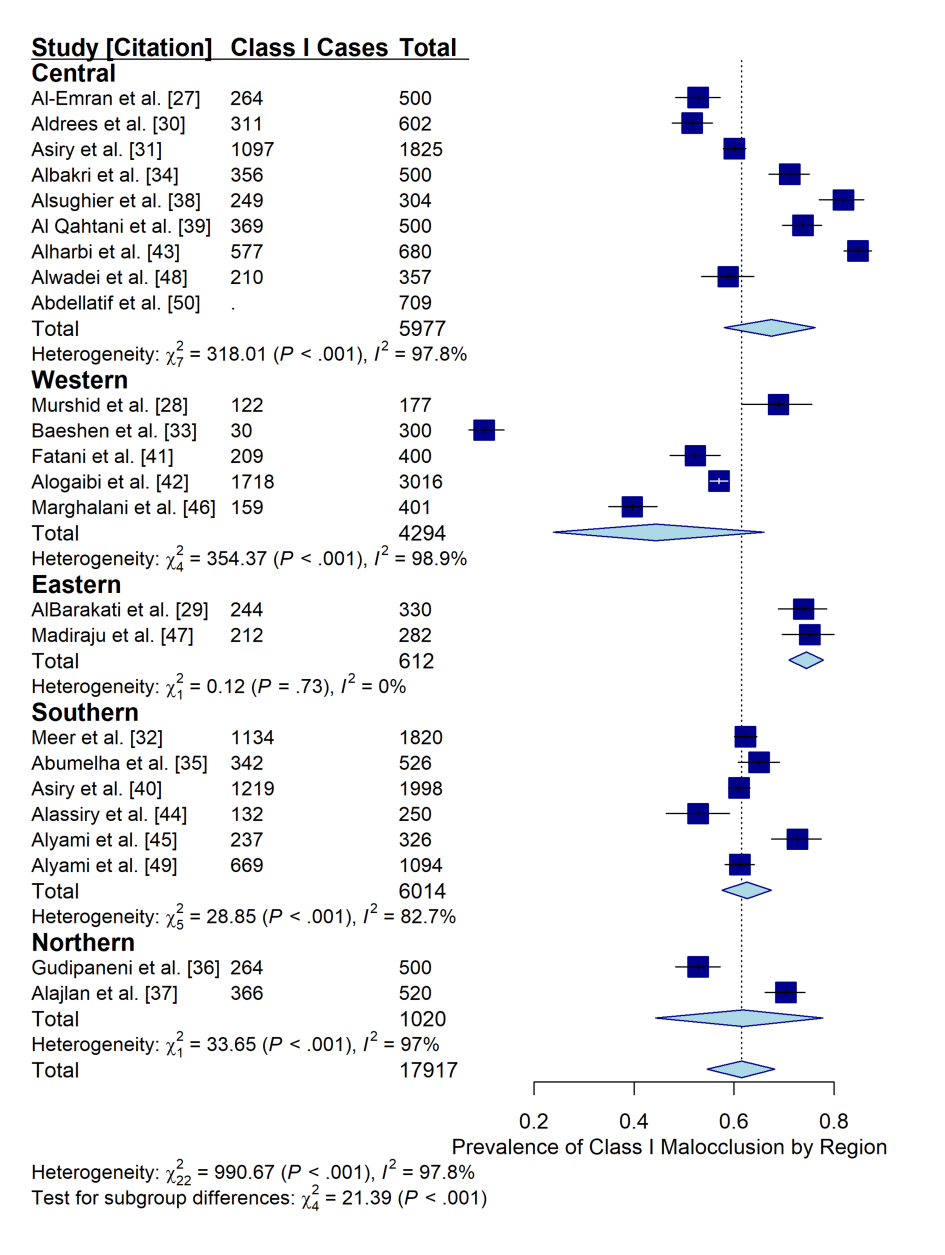
Subgroup analysis of Class I malocclusion prevalence stratified by geographic region (Central, Eastern, Northern, Southern, Western). The forest plot demonstrates significant heterogeneity between regions (p < 0.001), highlighting regional variations in malocclusion patterns across Saudi Arabia.

Subgroup analysis based on geographic region revealed significant variations in the prevalence of Class I malocclusion (p < 0.001). The Northern region reported the highest pooled prevalence, whereas the western region showed the lowest. This variability suggests that while Class I is the dominant malocclusion type nationally, its specific prevalence is influenced by regional demographic or environmental factors (Figure 7).

Impact of Study Setting (Robustness Check)

**Figure 8:**
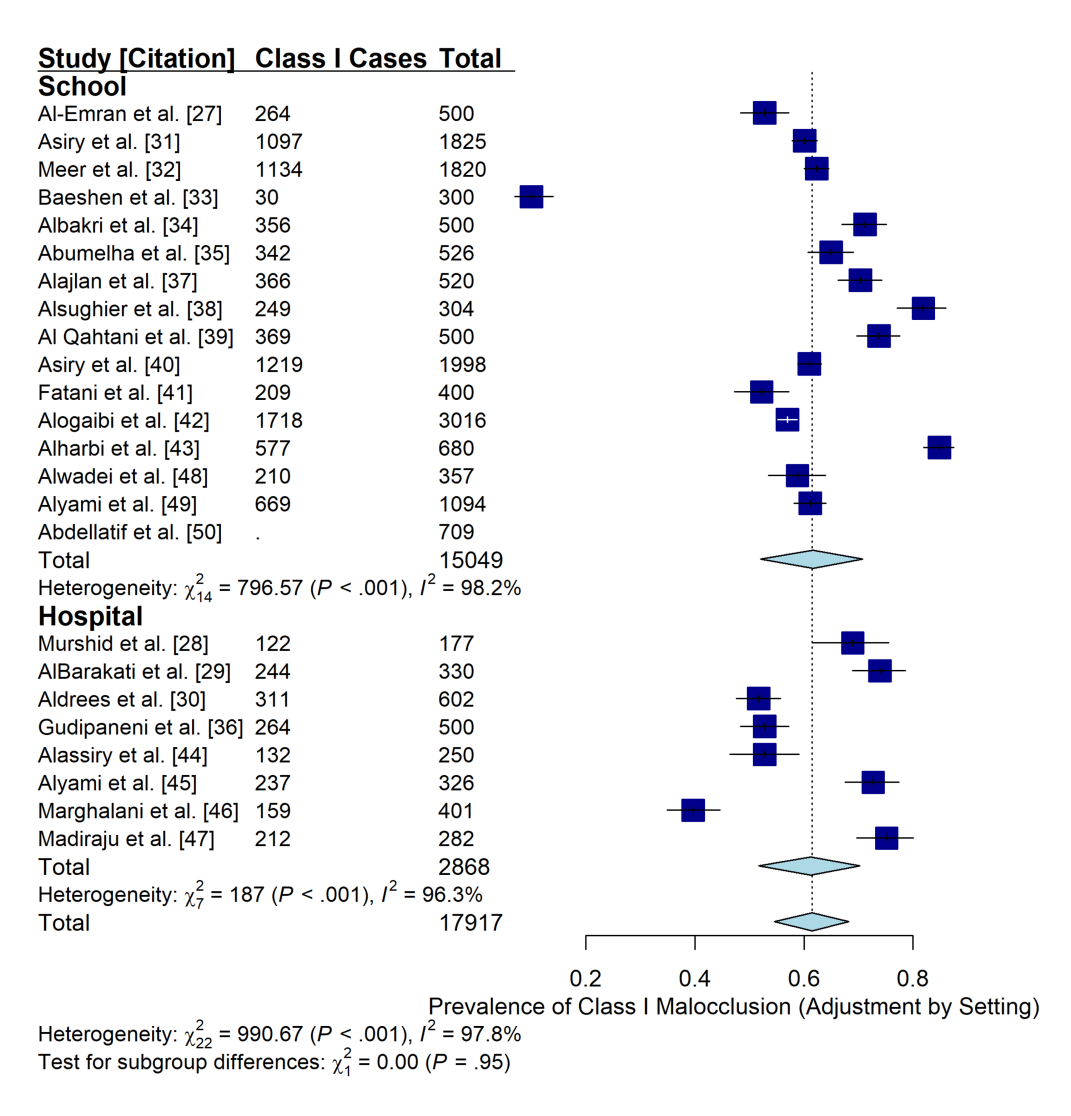
Robustness check comparing the prevalence of Class I malocclusion between school-based and hospital-based studies. The test for subgroup differences (p = 0.95) indicates that the study setting did not significantly bias the pooled prevalence estimate, supporting the generalizability of the findings.

To assess the impact of selection bias on the reported prevalence rates, a subgroup analysis was performed comparing school-based (population-based) studies with hospital/clinic-based studies. The analysis revealed no statistically significant difference in the pooled prevalence of Class I malocclusion between the two settings (p = 0.95). The pooled prevalence was 71.1% for both school- and hospital-based studies (Figure 8). This robustness check suggests that the high prevalence of malocclusion reported in hospital settings reflects the general population and is not merely an artefact of treatment-seeking behavior.

Temporal evolution (meta-regression)

To investigate potential temporal trends in the prevalence of malocclusion over the past three decades, a continuous random-effects meta-regression analysis was conducted with the Publication Year as the moderator. The analysis included 23 studies published between 1990 and 2024.

**Figure 9:**
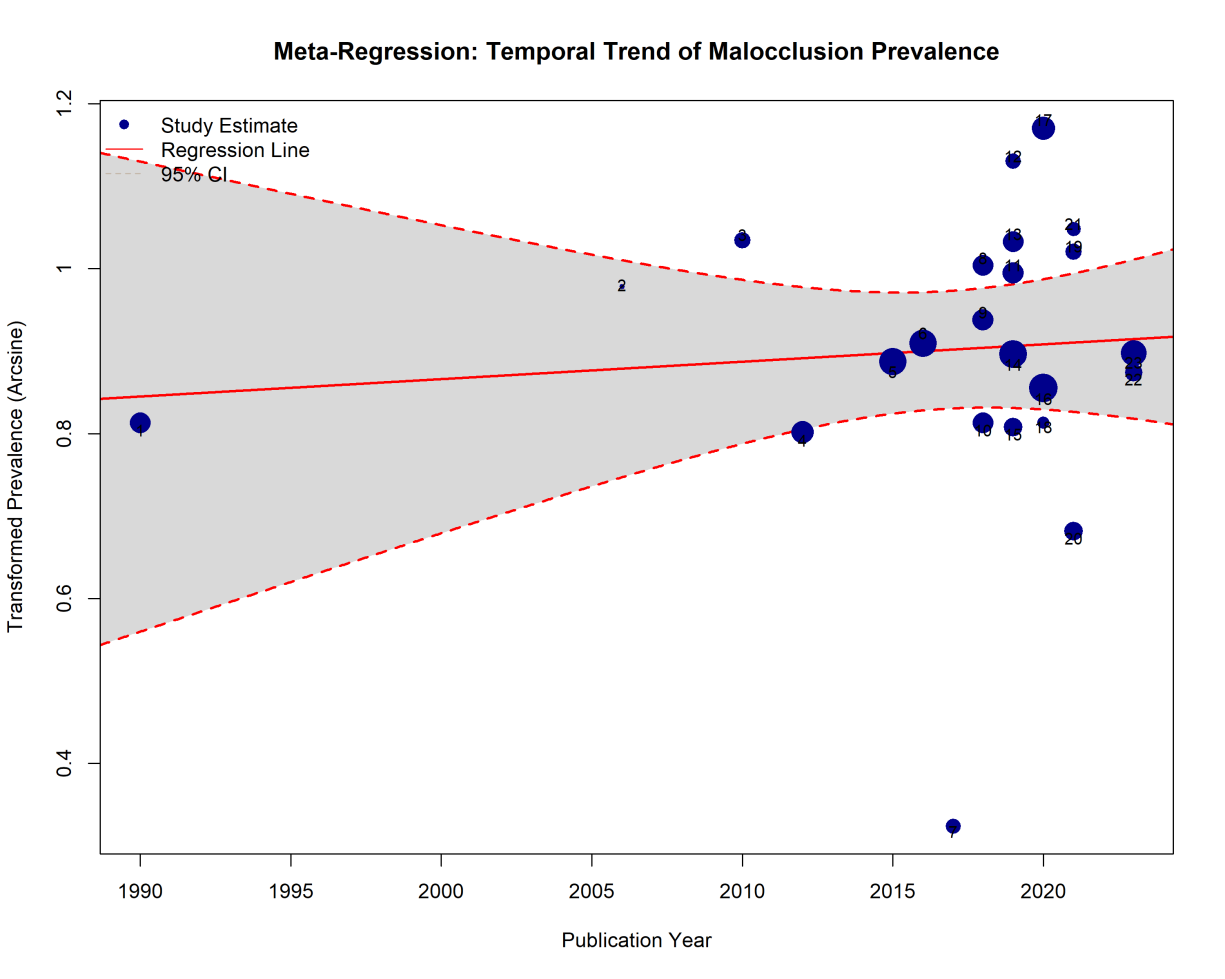
Meta-regression bubble plot illustrating the temporal trend of Class I malocclusion prevalence in Saudi Arabia from 1990 to 2024. Each blue circle represents a primary study, with the size of the circle proportional to the study's weight in the analysis. The solid red line represents the linear regression slope, and the dashed red lines indicate the 95% CIs. The flat slope (p = 0.69) indicates no significant change in prevalence over time.

The meta-regression model did not reveal a statistically significant linear relationship between the publication year and the prevalence of Class I malocclusion (β = 0.002; SE = 0.005; p = 0.69). The slope of the regression line was nearly flat, indicating that the reported prevalence of malocclusion in the Saudi population remained relatively stable over the 34-year period (Figure 9). Despite significant heterogeneity among the studies (Q = 977.3; p < 0.001), the year of publication accounted for 0.00% of the heterogeneity (R² = 0.00%), suggesting that factors other than time (e.g., regional differences and diagnostic criteria) are responsible for the observed variance in prevalence rates.

This stability suggests that while awareness and demand for orthodontic treatment may have increased, the actual epidemiological burden of malocclusion in the population has not changed significantly.

Sensitivity and publication bias analysis

Sensitivity Analysis

A "Leave-One-Out" sensitivity analysis was conducted to evaluate the robustness of the pooled prevalence estimate by iteratively removing one study at a time and recalculating the summary effect. The results demonstrated that no single study disproportionately influenced the overall pooled prevalence, as the recalculated estimates remained within the 95% CI of the meta-analysis (Table 3), confirming that the findings were robust and not driven by outliers or large-sample studies.

**Table 3:** Sensitivity analysis (Leave-One-Out Method). Evaluation of the stability of the pooled prevalence of Class I malocclusion when individual studies are omitted.

Study Omitted	Tau²	Tau	I² (%)	Pooled Prevalence (95% CI)
Al-Emran et al. [27]	0.0298	0.1725	97.8%	0.71 (0.55-0.85)
Murshid et al. [28]	0.0299	0.1728	97.9%	0.71 (0.54-0.85)
AlBarakati et al. [29]	0.0293	0.1711	97.8%	0.71 (0.54-0.85)
Aldrees et al. [30]	0.0296	0.1722	97.8%	0.71 (0.55-0.85)
Asiry et al. [31]	0.0301	0.1736	97.9%	0.72 (0.55-0.85)
Meer et al. [32]	0.0301	0.1736	97.9%	0.72 (0.55-0.85)
Baeshen et al. [33]	0.0137	0.1171	96.4%	0.74 (0.64-0.82)
Albakri et al. [34]	0.0296	0.1721	97.8%	0.71 (0.54-0.85)
Abumelha et al. [35]	0.0301	0.1734	97.9%	0.71 (0.55-0.85)
Gudipaneni et al. [36]	0.0298	0.1725	97.8%	0.72 (0.55-0.85)
Alajlan et al. [37]	0.0297	0.1723	97.8%	0.71 (0.54-0.85)
Alsughier et al. [38]	0.0275	0.1660	97.7%	0.70 (0.54-0.84)
Al Qahtani et al. [39]	0.0293	0.1711	97.8%	0.71 (0.54-0.85)
Asiry et al. [40]	0.0301	0.1736	97.9%	0.72 (0.55-0.85)
Fatani et al. [41]	0.0297	0.1724	97.9%	0.72 (0.55-0.85)
Alogaibi et al. [42]	0.0300	0.1733	97.8%	0.72 (0.55-0.85)
Alharbi et al. [43]	0.0265	0.1628	97.3%	0.70 (0.55-0.84)
Alassiry et al. [44]	0.0298	0.1725	97.9%	0.72 (0.55-0.85)
Alyami et al. [45]	0.0294	0.1716	97.8%	0.71 (0.55-0.85)
Marghalani et al. [46]	0.0277	0.1665	97.7%	0.72 (0.56-0.85)
Madiraju et al. [47]	0.0291	0.1705	97.8%	0.71 (0.54-0.85)
Alwadei et al. [48]	0.0301	0.1735	97.9%	0.72 (0.55-0.85)
Alyami et al. [49]	0.0301	0.1736	97.9%	0.72 (0.55-0.85)
Abdellatif et al. [50]	0.0287	0.1695	97.8%	0.71 (0.55-0.85)
Total (All Included)	0.0287	0.1695	97.8%	0.71 (0.55-0.85)

Publication Bias

**Figure 10:**
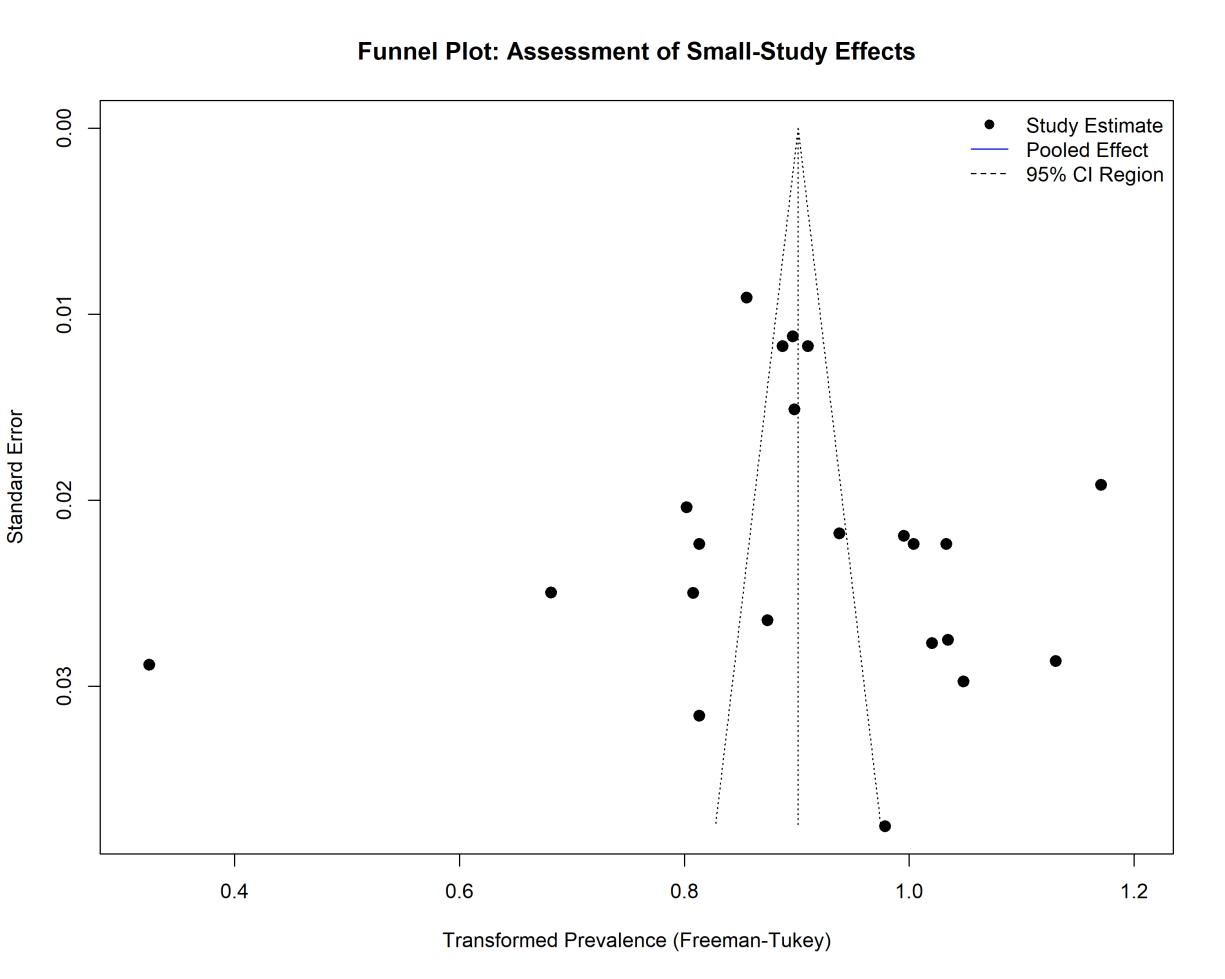
Funnel plot for the assessment of publication bias. The x-axis represents the transformed prevalence, and the y-axis represents the standard error (precision). The symmetrical distribution of studies (black dots) within the funnel indicates a low risk of publication bias.

The potential for publication bias (small study effects) was assessed both visually and statistically. Visual inspection of the funnel plot revealed a generally symmetrical distribution of studies around the pooled effect estimate, suggesting no severe publication bias (Figure 10). This observation was statistically confirmed using Egger’s Linear Regression Test, which showed no significant asymmetry (t = 0.29; p = 0.78). Similarly, Begg’s rank correlation test indicated no significant correlation between the effect size and sample size (z = 0.87; p = 0.38).

**Figure 11:**
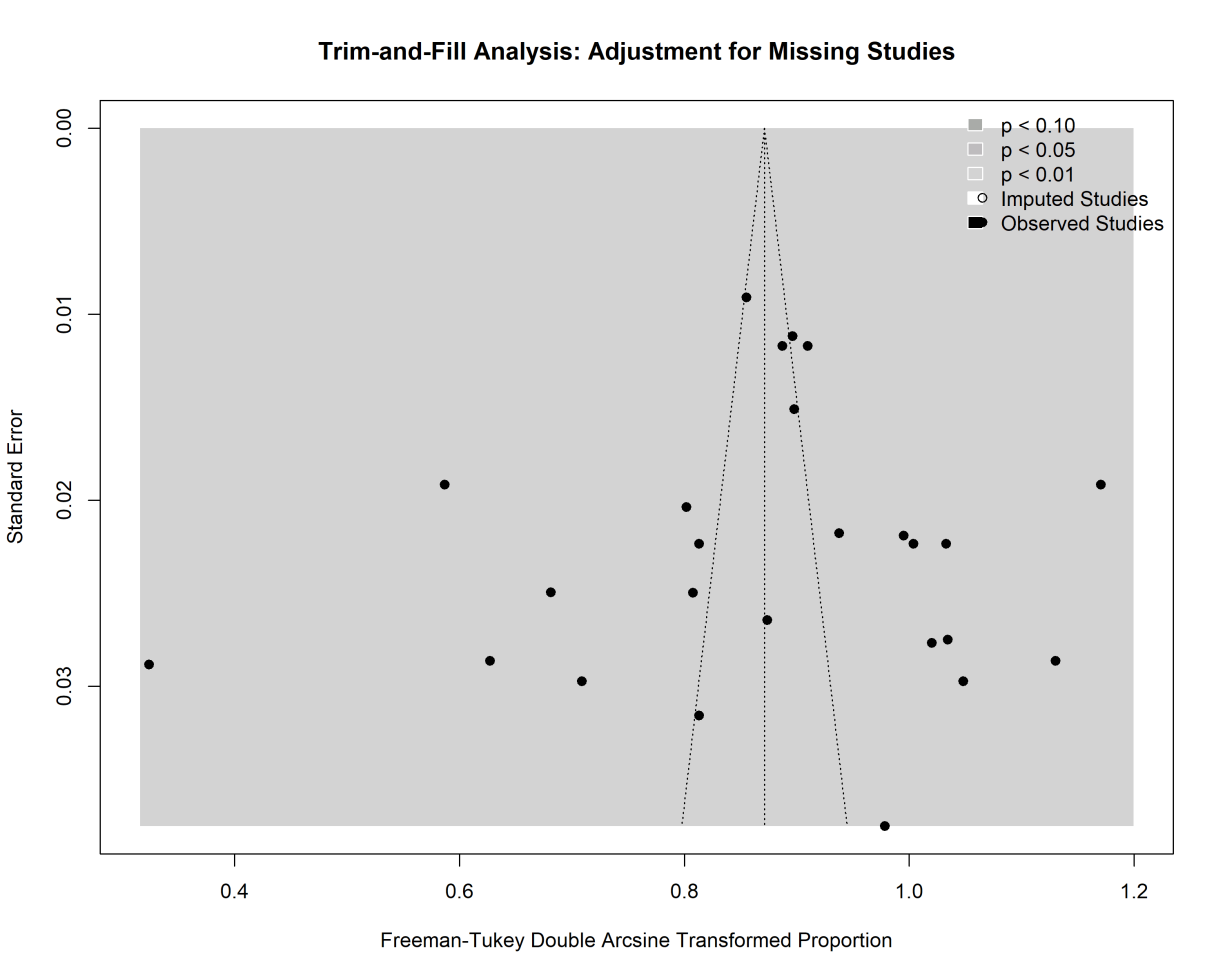
Trim-and-fill funnel plot. Open circles represent the imputed "missing" studies required to make the funnel symmetrical. The minimal shift in the pooled estimate after imputation suggests that the results are robust against potential publication bias.

The trim-and-fill method was applied to further validate these findings. The analysis identified three missing studies on the left side of the funnel plot. After imputing these theoretically missing studies, the adjusted pooled prevalence decreased slightly from 71.1% (original random effects) to 58.6% (trim-and-fill adjusted). However, the 95% CIs of the adjusted estimate (51.6%-65.4%) overlapped significantly with the original estimate, indicating that even if publication bias exists, its impact on the overall conclusion is minimal (Figure 11).

Certainty of evidence and sample size sufficiency

Certainty of Evidence (GRADE)

The overall certainty of the evidence was assessed using the GRADE approach. The evidence for the prevalence of malocclusion and dental anomalies was graded as moderate. While the included studies were observational (cross-sectional), which start at low certainty, the evidence was upgraded due to the large magnitude of effect (consistent high prevalence across regions) and the dose-response gradient observed in specific associations (e.g., age and root dilaceration). However, the rating was constrained by the high statistical heterogeneity (I² > 90%) observed in the pooled analysis, although this is inherent to proportional meta-analyses of prevalence studies (Table 4).

**Table 4:** Summary of certainty of evidence (GRADE) and sample size sufficiency analysis.

Parameter	Value	Interpretation
Pooled Prevalence	71.1%	High burden of malocclusion.
Heterogeneity (I²)	97.8%	High variability between studies (expected in prevalence data).
Required Sample Size (OIS)	14,213	Calculated to achieve 80% power.
Actual Sample Size	17,917	Sufficient. Exceeds requirement.
Evidence Grade	Moderate	Robust estimate despite heterogeneity.

Sample Size Sufficiency (Optimal Information Size)

To determine whether the cumulative sample size of the included studies was sufficient to draw definitive conclusions, a TSA approach was used to calculate the OIS. Based on a pooled prevalence of 71.1%, statistical power of 80%, type I error rate (α) of 5%, and adjustment for observed heterogeneity (I² = 97.8%), the required sample size was calculated to be 14,213 participants.

The total cumulative sample size of the 53 studies included in this systematic review was 17,917 participants, which exceeded the calculated OIS. This confirms that the current body of evidence has sufficient statistical power to reliably estimate the prevalence of dentofacial characteristics in the Saudi population. Further simple prevalence studies are unlikely to change the overall conclusions, and future research should prioritize the investigation of risk factors, etiology, and intervention outcomes.

Discussion

This systematic review and meta-analysis provide the most comprehensive synthesis of epidemiological data on dentofacial characteristics in Saudi Arabia to date, pooling data from 53 studies involving 17,917 participants over a 42-year period (1983-2025). The findings revealed a substantial burden of malocclusion and dental anomalies within the Saudi population, with significant regional variations that necessitate targeted public health interventions.

Prevalence of Malocclusion

The pooled prevalence of Angle’s Class I malocclusion was found to be 71.1%, establishing it as the predominant occlusal pattern in the Saudi population, which aligns with global epidemiological trends reported in other Arab and Middle Eastern populations, where Class I malocclusion consistently ranges between 60% and 75% [1,3]. However, the prevalence of Class II (16.3%) and Class III (9.8%) malocclusions in our study was notably higher than the global averages for Caucasian populations (Class II: ~10%; Class III: ~3-5%) [4]. This discrepancy may be attributed to specific cephalometric features inherent to the Saudi ethnic background, such as bimaxillary protrusion and a tendency towards a convex profile, as highlighted in previous cephalometric studies [6,30].

Dental crowding was the most frequent specific malocclusion trait, affecting 39.7% of the population. This high prevalence is consistent with the global secular trend of increasing crowding due to the reduction in human jaw size without a corresponding decrease in tooth size, a phenomenon often linked to the consumption of softer, processed diets in modern societies [4,42]. The substantial prevalence of increased overjet (28.4%) and deep overbite (16.3%) further underscores the need for early orthodontic screening, as these traits are known risk factors for dental trauma and temporomandibular joint disorders [5].

Prevalence of Dental Anomalies

The prevalence of dental anomalies in the Saudi population showed marked variability, ranging from 11.2% to 67.8%. Hypodontia was a significant finding, with a pooled prevalence of 4.6% to 25.7% depending on the region. This range is considerably higher than the 3.2%-7.6% prevalence reported in most European and North American populations [7,52]. The high rate of hypodontia, particularly agenesis of the mandibular second premolars, may indicate a genetic predisposition within the Saudi population, potentially influenced by the high rates of consanguinity in certain regions [2,7].

Root dilaceration emerged as a surprisingly common anomaly, particularly in the Eastern Province (30.2%) [53]. While often considered an incidental finding, its high prevalence has critical clinical implications for endodontic and orthodontic treatments, increasing the risk of complications such as root resorption and incomplete obturation.

Regional and Temporal Trends

Subgroup analysis revealed significant regional disparities (p < 0.001), with the Northern region reporting the highest prevalence of malocclusion traits. This variation could be driven by a combination of genetic clustering in tribal communities and disparities in access to early preventive dental care [36,49]. The meta-regression analysis indicated no significant temporal trend (p = 0.69) in the prevalence of malocclusion from 1990 to 2024. This stability suggests that the burden of malocclusion has remained constant over the last three decades, implying that despite improvements in dental healthcare infrastructure in Saudi Arabia, preventive measures targeting malocclusion etiology (e.g., interceptive orthodontics and space maintenance) have not yet yielded a population-level reduction in prevalence [72].

Robustness and Quality of Evidence

A key strength of this review was the rigorous assessment of methodological quality and publication bias. The "Leave-One-Out" sensitivity analysis confirmed that the pooled estimates were stable and not driven by any single-outlier study. Furthermore, the adjustment analysis comparing school-based and hospital-based studies showed no significant difference (p = 0.95), debunking the common assumption that hospital-based studies overestimate prevalence due to selection bias. This suggests that in the context of Saudi Arabia, where dental screening is often integrated into hospital visits, hospital records may serve as a reliable proxy for population health. The extensive cumulative sample size (N > 17,000) provided sufficient statistical power to draw definitive conclusions, satisfying the OIS requirements.

Limitations

The high statistical heterogeneity (I² > 90%) observed is inherent to proportional meta-analyses of prevalence studies and reflects the diversity in diagnostic criteria (e.g., IOTN vs. Angle’s classification) and age groups across primary studies. Additionally, while we assessed regional variations, we could not account for specific tribal or genetic affiliations because of the lack of granular data in the primary studies. Finally, most of the included studies were cross-sectional, which precluded the establishment of causal relationships between risk factors and malocclusion.

Future Directions

Future research should move beyond simple prevalence survey studies. Longitudinal cohort studies are recommended to track the progression of malocclusion from primary to permanent dentition. Genetic association studies are also needed to investigate the high rates of hypodontia and Class III malocclusion in specific Saudi subpopulations. Implementation research may evaluate the cost-effectiveness of national school-based orthodontic screening programs, particularly in the high-prevalence Northern and Southern regions.

## Conclusions

The Saudi population exhibits a high burden of malocclusion and specific dental anomalies, particularly hypodontia and root dilaceration, with distinct regional patterns of prevalence. The stability of these rates over 30 years highlights the critical need for a shift from curative to preventive orthodontic public health strategies.
